# The NCI Cancer Tissue Engineering Collaborative Research Program is a highly interdisciplinary and focused community

**DOI:** 10.1016/j.isci.2021.102441

**Published:** 2021-04-30

**Authors:** Nastaran Zahir, Jennifer Couch

**Affiliations:** 1 Division of Cancer Biology, National Cancer Institute, National Institutes of Health, Rockville, MD, USA

**Figure:**
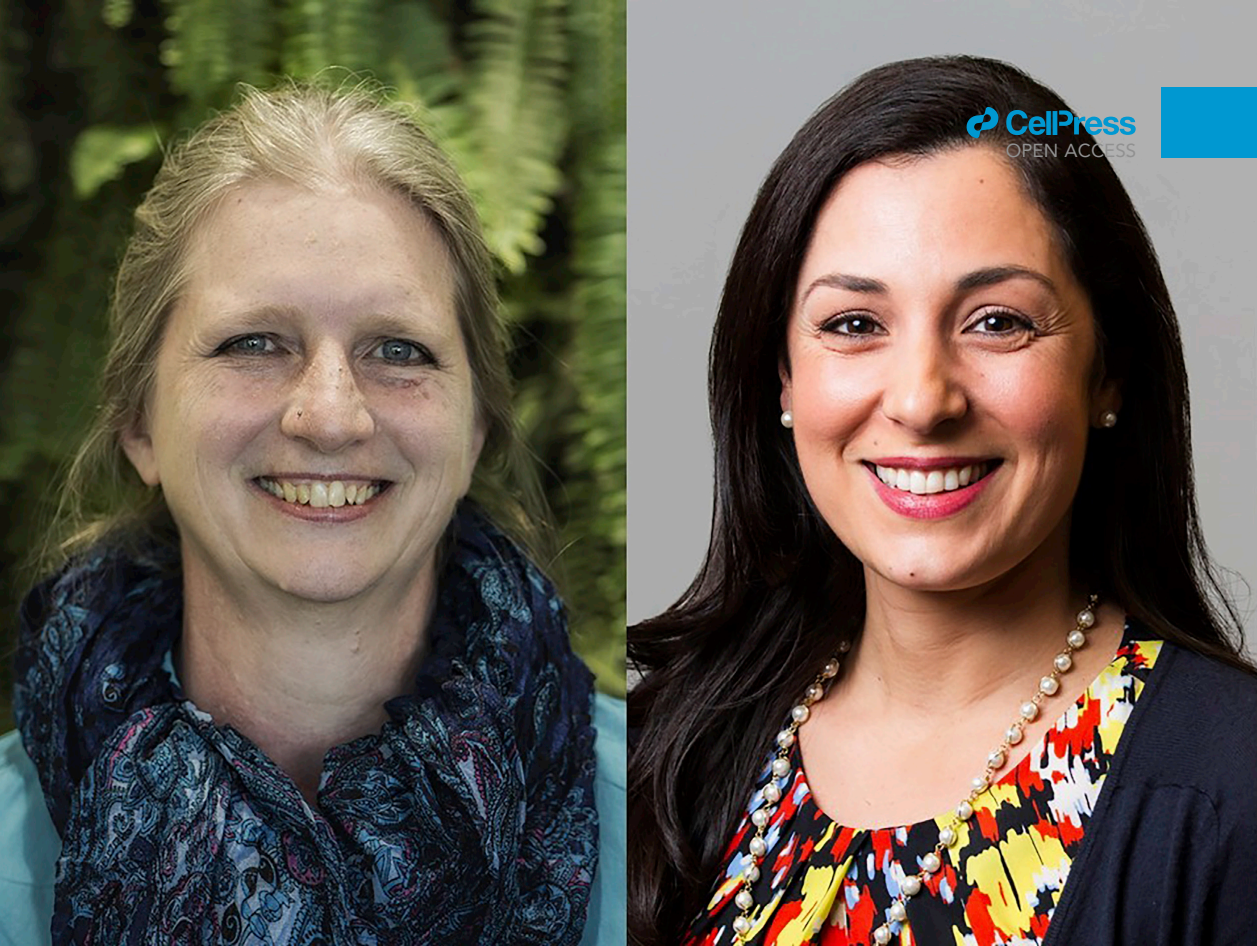
Jennifer Couch (left) and Nastaran Zahir (right).

This rapidly developing field is highly collaborative, sharing insights and approaches with each other and with the NCI Physical Sciences-Oncology Network investigators.

There are opportunities provided for the researchers in the Cancer TEC program to meet on a regular basis to discuss their platforms and share challenges faced with aspects related to the technology development, cancer tissue modeling, or testing of drugs in the systems.

The Cancer Tissue Engineering Collaborative (Cancer TEC) is a unique research program supported by the National Cancer Institute (NCI) developing and characterizing biomimetic tissue-engineered platforms meticulously designed to study cancer as a complex, adaptive system. These engineered three-dimensional systems fill a critical gap in the cancer modeling continuum, combining sophisticated biological systems which closely mimic the cancer and its surrounding microenvironment with the ability to manipulate, quantify, and monitor different parameters. The many disciplines needed to facilitate research of this caliber make the program inherently a highly interdisciplinary and focused community.

## Proximity

### How did Cancer TEC originate? What is unique about the teams involved?

The idea for the program originated from a growing appreciation of the importance of understanding the physical properties of cancer, through research supported by the NCI Physical Sciences-Oncology Network (PS-ON). Furthermore, the program was also an opportunity to leverage advances made in fields such as regenerative medicine where engineered systems were beginning to be developed. In 2012–2015, the NCI held a series of strategic workshops convening researchers across very different fields such as tissue engineering, developmental biology, cancer biology, and computational modeling to explore opportunities for developing physiologically relevant and controlled tissue-engineered systems for cancer. The workshop reports featuring these cross-disciplinary insights are available at https://physics.cancer.gov/report/. A funding opportunity was then issued by the NCI in 2016 for enabling biomimetic tissue-engineered technologies for cancer research, which is still active today.

There are currently 16 funded projects in the Cancer TEC Research Program, affiliated with the NCI PS-ON. Cancer TEC researchers are creating systems to study a wide variety of cancer questions, mimicking multiple cancer types including brain, breast, colon, skin, ovary, pancreas, prostate, and thyroid. These systems enable highly controlled investigation of key cancer mechanisms in tumor biology and progression, as well as patient drug response. These primarily three-dimensional (3D) systems include biomaterials, incorporate multiple cell types, and include physical and chemical factors of the tumor microenvironment necessary to control, perturb, and study cancer development and response in a realistic way. Cancer TEC investigators use a wide variety of state-of-the-art microfluidics, polymer physics, dynamic flow pattering and perfusion, and 3D printing approaches to design and build these systems. This rapidly developing field is highly collaborative, sharing insights and approaches with each other and with the NCI Physical Sciences-Oncology Network investigators.

More information about the Cancer TEC research program and the funded teams can be found at https://cancer.gov/tec.

### What are other programs which are close to the Cancer TEC program which will be instrumental for this program to work at its best?

NCI has many programs that can help to advance the complexity of the biomimetic tissue-engineered platforms and increase impact of the Cancer TEC Research Program. The PS-ON helps to elucidate the impact of physical and mechanical properties of the tumor microenvironment on disease progression and how those factors couple with chemical factors to change cell phenotype, cell-state transitions and alter thermodynamic equilibrium of the system. The Cancer Systems Biology Consortium advances the mechanistic understanding of cancer complexity through the explicit integration of experimental biology and computational and mathematical methods to build predictive models of cancer that are tested or validated in a disease-relevant context. The Patient-Derived Models of Cancer (PDMC) tests and compares distinct patient-derived models developed from common patient samples with the objective of improving the understanding of the strengths and limitations of different patient-derived models as representatives of human tumors. The PDMC program is undertaking systematic studies of patient-derived model’s evolution related to intrinsic tumor factors and microenvironmental pressures. Improved patient-derived models for cancer may inform the development of novel cancer therapies.

The Human Tumor Atlas Network is constructing 3D atlases of the cellular, morphological, and molecular features of human cancers over time. The Early Detection Research Network provides insights about the early biomarkers of cancer risk and initiation and can help to identify tumor microenvironmental factors that could drive early disease formation. Finally, the NIH National Center for Advancing Translational Sciences program has the Tissue Chip (aka Microphysiological Systems) program that really helped to kick-start the early development of these microfluidic platforms, first for accurately modeling the structure and function of human organs to help predict drug safety in humans more rapidly and effectively. During the program’s inception, it focused on developing physiologically relevant models for toxicity testing. The current focus of the program is on disease modeling and efficacy testing including clinical trials on chip.

## Methods

### The Cancer TEC research program will doubtlessly lead to a number of high-profile research articles. What about other deliverables, such as improved infrastructure for sharing models across labs, or initiatives to increase reproducibility of research outputs?

There are opportunities provided for the researchers in the Cancer TEC program to meet on a regular basis to discuss their platforms and share challenges faced with aspects related to the technology development, cancer tissue modeling, or testing of drugs in the systems. These meetings in addition to opportunities for supplemental funding to support collaborative projects can help to cross-validate platforms across laboratories. This is an important step in the development and widespread utility of the platforms across the cancer research community. Furthermore, we encourage collaborations with other groups who are not directly funded through the Cancer TEC program, with the aim of increasing the diversity of approaches and diversity of researchers who are actively participating in the program.

## Final thoughts

### What is the added value of this collaboration versus having the same question approached by different communities (e.g. cancer biologists and tissue engineers) separately?

Tissue engineers have been tackling questions of regenerative medicine for decades, in collaboration with developmental biologists and other biologists with tissue-level specificity. Disease modeling, such as for cancer, came later.

Technology development in a cancer-relevant context of use is driven by specific questions in cancer biology or clinical oncology. For example, can tortuous blood flow in the tumor microenvironment be mimicked in a microfluidic platform to emulate the diffusion of nutrients that would be seen in a tumor? Or, can such platforms reproduce the heterogeneity of drug response with emergence of resistant cell populations that would be observed in a patient with cancer? These are some questions that can be asked that drive the technology development forward in collaboration with cancer biologists and help to move the field beyond traditional murine animal model systems that may not always mimic tumor evolution or response to treatment.

In addition, these collaborations can elicit further understanding of when the platforms are useful alone or in parallel with other experimental model systems for cancer. Clinician scientists who are part of the Cancer TEC teams help to ensure that the model systems are relevant to human cancer progression and potentially move some of their applications toward the clinic.

